# Distal Radius Reconstruction With Non-vascularized Proximal Fibular Autograft After en-Bloc Resection of Giant Cell Tumors: A Retrospective Study

**DOI:** 10.7759/cureus.88840

**Published:** 2025-07-27

**Authors:** Mir Shahid ul Islam, Vishal Sidhu, Naim Akbar, Altaf Hussain, Najamul Huda, Goduguluri Naveen Kumar Choudary, Mohd Faizullah, Mohd Ashar ul Huda

**Affiliations:** 1 Orthopaedics, Venkateshwara Institute of Medical Sciences, Gajraula, IND; 2 Medicine and Surgery, Jawaharlal Nehru Medical College, Aligarh, IND

**Keywords:** autologous fibula graft, distal radius gct, gct, msts score, reconstruction after tumor resection

## Abstract

Background

Giant cell tumors (GCTs) of the distal radius present significant reconstructive challenges following wide resection. Proximal fibular autograft reconstruction has been a biologically compatible solution, particularly valuable in resource-limited settings. This study evaluates clinical and functional outcomes of this technique.

Materials and methods

We retrospectively analyzed 14 patients (10 females, four males; mean age 30.9±5.4 years) with eight Campanacci grade II (57.1%) and six patients of grade III (42.9%) distal radius GCTs treated with en-bloc resection and proximal fibular autograft reconstruction. Outcomes were assessed using Musculoskeletal Tumor Society (MSTS) scores, range of motion (ROM) measurements, grip strength, and complication rates.

Results

Mean MSTS score was 20.6±2.9 (range 15-24). Mean wrist ROM included flexion 28.2°±7.0°, extension 20.7°±6.1°, radial deviation 10.1°±3.3°, and ulnar deviation 10.0°±4.4°. Forearm rotation was well preserved (supination 45.4°±14.7°, pronation 49.6°±10.5°). Grip strength averaged 60.9%±7.4% of the contralateral side. Complications included seven fibula-carpal subluxations (50%), four fibula-ulnar diastases (28.6%), one non-union (7.1%), and one transient foot drop (7.1%). The majority of our patients (n=11, 78.5%) were either pain-free or had mild pain, while only three patients (21.5%) had moderate pain, with none having intolerable pain. All patients achieved independence in activities of daily living. Among seven manual laborers, four returned to their original work while three required modifications; all non-manual workers resumed pre-operative activities.

Conclusion

En-bloc resection and proximal fibular autograft reconstruction for distal radius GCTs provide satisfactory functional outcomes with acceptable complication rates. While wrist mobility shows some limitation compared to normal values, most patients achieve good functional recovery and return to daily activities

## Introduction

Giant cell tumors (GCTs) are benign yet locally aggressive neoplasms that predominantly occur in the epiphysis of long bones, with peak incidence between the ages of 20 and 40 years [1]. While the distal radius is the third most common site for GCTs (after the distal femur and proximal tibia), tumors in this location often exhibit more aggressive behavior and a higher propensity for recurrence [2, 3].

The primary treatment strategies for distal radius GCTs include extended curettage with adjuvants (e.g., phenol, cryotherapy, or bone cement) or en bloc wide resection followed by reconstruction. The choice depends on the tumor stage (Campanacci classification) and associated risks [4, 5]. Extended curettage preserves the native joint but carries a higher recurrence risk, whereas wide resection and arthrodesis reduce recurrence rates at the expense of wrist joint function and normal anatomy [6]. Reconstruction after wide resection poses significant challenges. Current reconstruction techniques include structural autografts (vascularized or non-vascularized), allografts, ulnar centralization with wrist arthrodesis, and prosthetic replacement [7, 8]. Among these, proximal fibular autografts have been widely used for reconstruction, though outcomes vary in terms of functional recovery and complications [9-11].

This study evaluates the outcomes of Campanacci grade II and III GCT of the distal radius managed with wide resection and reconstruction using autogenous non-vascularized osteoarticular fibular grafts, focusing on oncological control, functional restoration, and complications.

## Materials and methods

Study design and setting

This retrospective study was conducted in the department of orthopaedics at a tertiary care hospital between June 2024 and November 2024. Institutional Ethical Committee approval was obtained (Reference No. VIMS/IEC/2024/22) before data collection.

Patient selection

Hospital records were reviewed to identify patients who underwent surgical treatment for GCT of the distal radius between January 2017 and October 2023. Of the 19 patients initially identified, 14 met the inclusion criteria and were included in the final analysis. Written informed consent was obtained from all participants for both surgical intervention and use of anonymized clinical data, following the Declaration of Helsinki principles.

Inclusion and exclusion criteria

**Figure 1:**
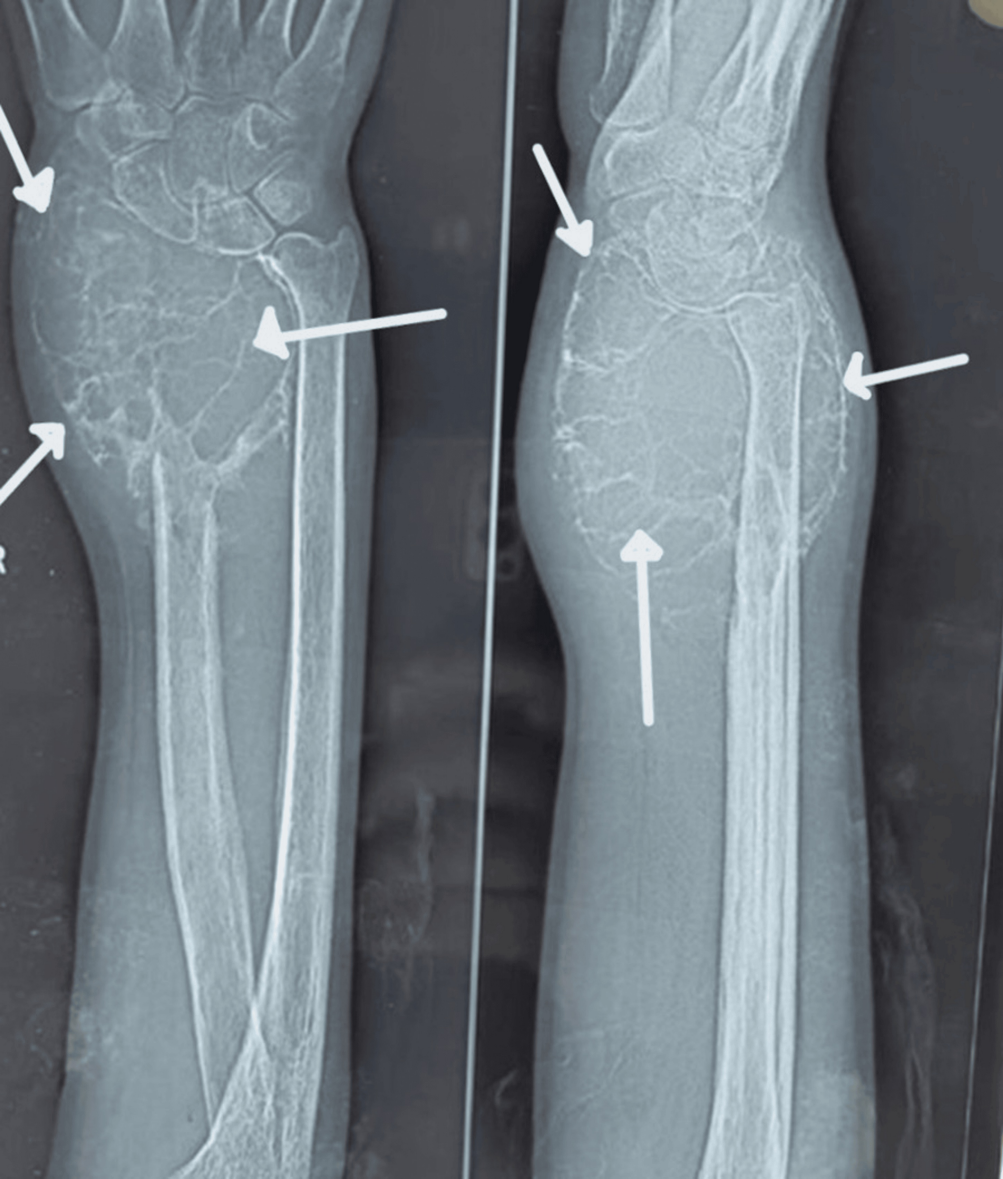
Pre-operative radiograph Shows an expansile, lobulated, lytic lesion involving the distal radius (white arrows) with cortical destruction Campanacci Grade III Giant cell tumor (GCT) in a 31-year-old patient.

Histologically confirmed and Campanacci grade II or III GCT of the distal radius (Figure 1) treated in our institution with a minimum follow-up of one year were included, while patients who were lost to follow-up or unwilling to participate, or with incomplete data and Campanacci grade I lesions were excluded. Grade I lesions were managed with extended curettage and void filling with bone cement/bone graft.

Data collection

Medical records were analyzed for demographic characteristics (age, gender, laterality), tumor characteristics (Campanacci grade, preoperative biopsy results, postoperative histopathology including margin status), and surgical details (surgical approach, size of resected tumor, and graft length).

Functional outcomes were evaluated using three standardized measures: (1) the Musculoskeletal Tumor Society (MSTS) scoring system [12], (2) the visual analogue scale (VAS) for pain assessment, and (3) objective measurements including range of motion (ROM) (wrist flexion-extension, radial-ulnar deviation) and grip strength (measured using a calibrated dynamometer, expressed as percentage of contralateral side).

Serial radiographs were evaluated for bony union at the fibula-radius junction, maintenance of fibulocarpal alignment (measuring subluxation/dislocation), fibuloulnar joint integrity or diastasis, implant failure, and evidence of local recurrence.

Surgical technique

**Figure 2:**
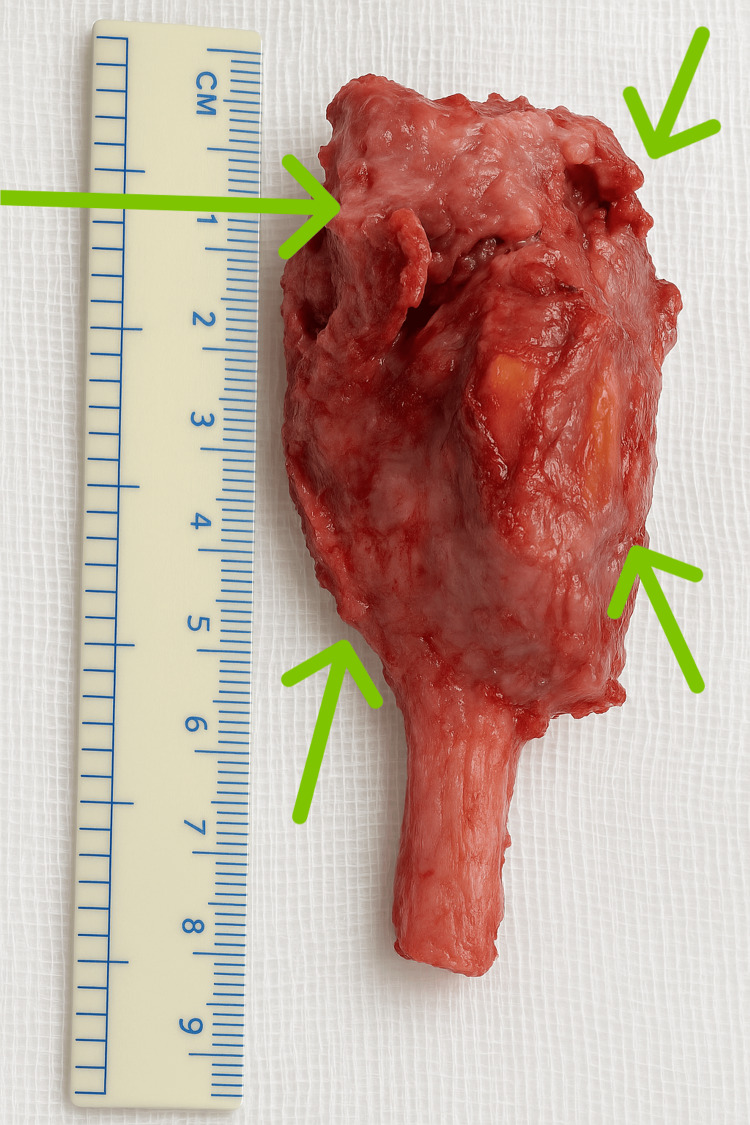
Surgically excised 10.3 cm GCT specimen (green arrows) following wide resection GCT: Giant cell tumor

All procedures were performed under general anesthesia with patients in the supine position under tourniquet control by a single surgical team. Through a volar approach, we carefully dissected and protected the neurovascular bundle before performing extraperiosteal tumor resection to achieve en bloc removal without capsular violation (Figure 2). Tumor-free margins of 3-5 cm were obtained based on preoperative imaging assessment, with the proximal resection margin sent for intraoperative frozen section confirmation. The wrist joint capsule was preserved where possible without compromising tumor margins.

**Figure 3:**
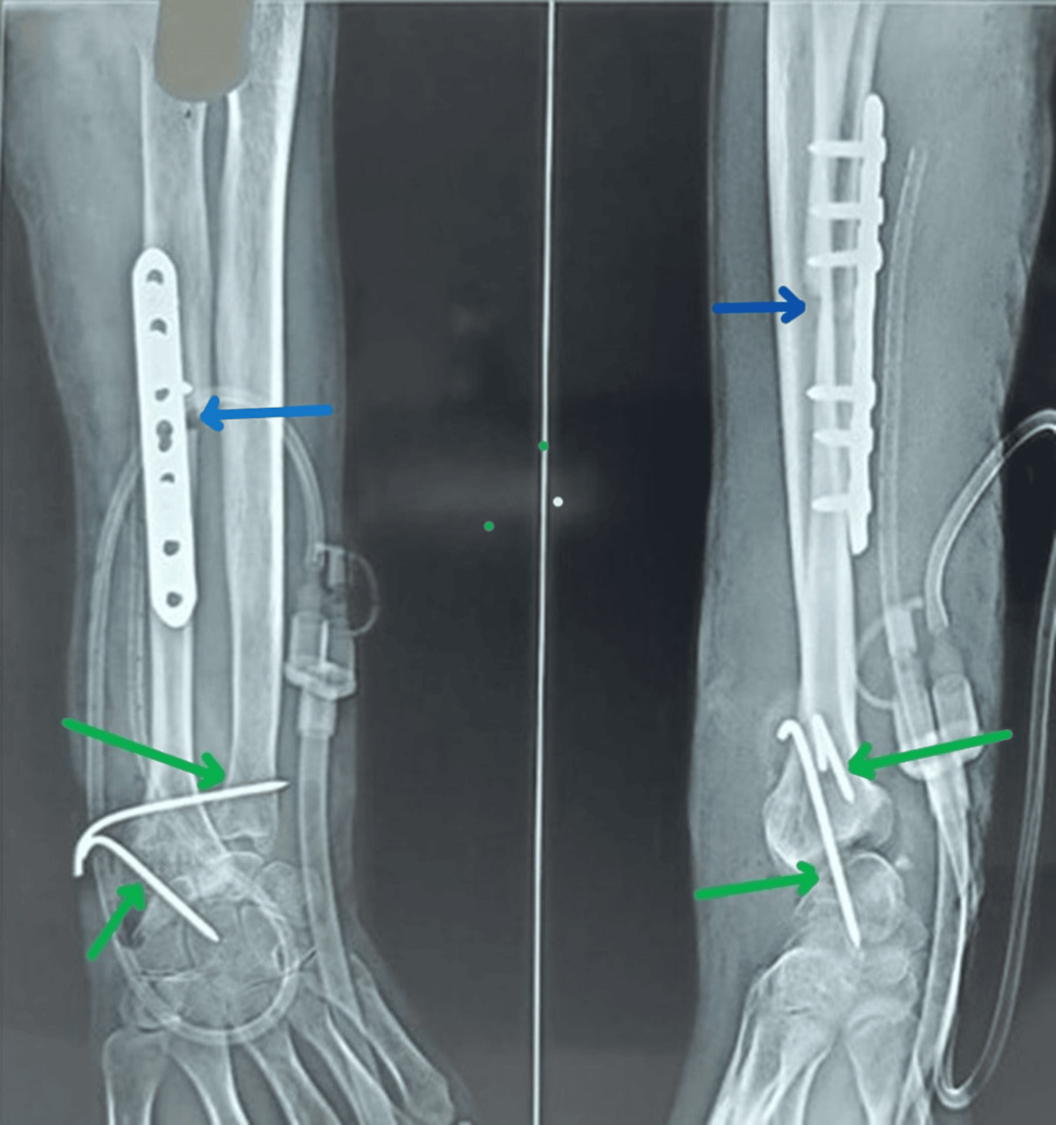
Post-operative radiograph Demonstrates successful tumor resection with fibulo-radial fixation using a 3.5 mm plate (blue arrows) and K-wire stabilization of the fibulo-carpal and fibulo-ulnar joints (green arrows).

For reconstruction, the ipsilateral proximal fibula was harvested, including its articular surface, through a lateral approach, taking care to identify and protect the common peroneal nerve. The fibular graft was intentionally cut 1 cm longer than the measured defect to accommodate potential measurement errors. We preserved a small cuff of soft tissue attached to the fibular head for subsequent soft tissue reconstruction. The attachments over the fibular head (lateral collateral ligament and biceps tendon) were approximated and sutured to the proximal tibia through drill holes. The retained soft tissue cuff over the graft was sutured to the wrist joint capsule and secured to the radius using a 3.5 mm compression plate, with temporary stabilization of the fibulocarpal and fibuloulnar joints using K-wires (removed at six weeks postoperatively) (Figure 3). Patients were immobilized in a long arm plaster backslab for six weeks, following which active and assisted range of motion exercises were initiated.

## Results

Fourteen patients underwent wide resection of distal radius giant cell tumors with reconstruction using non-vascularized proximal fibular autografts. The cohort comprised 10 females (71.4%) and four males (28.6%), with a mean age of 30.93 ± 5.41 years (range 19-42 years). Right-sided involvement was observed in eight cases (57.1%), and left-sided involvement in six cases (42.9%). All patients were right-hand dominant individuals. According to the Campanacci classification, eight cases (57.1%) were grade II and six (42.9%) were grade III. None had metastasis or a pathological fracture at presentation. The study had a mean follow-up period of 39.86 ± 19.2 months (range 13-71 months), with all surgical procedures following a standardized protocol. The average length of the excised radius was 10.74 ± 1.47 cm (9-13 cm) (Table 1). Resection margins were maintained at 3-5 cm of normal bone, as preoperatively determined by MRI assessment.

**Table 1:** Demographics, tumor grade, follow-up duration, and excised radius length in included patients

S.No.	Age (years)	Sex	Side	Campanacci grade	Follow-up Duration (months)	Length of radius excised (cm)
1	32	F	R	III	71	12
2	27	F	L	II	62	13
3	34	F	R	III	58	10.3
4	19	M	R	II	54	12.5
5	31	F	L	III	51	11.3
6	42	F	R	II	46	13
7	37	F	L	II	44	9.5
8	28	M	R	II	44	11
9	29	F	R	III	42	9
10	33	F	R	II	21	9.4
11	35	M	L	III	19	10.2
12	30	F	R	II	17	11
13	29	m	L	II	16	9.1
14	27	f	L	III	13	9

Functional scores and range of motion (ROM)

Mean MSTS score was 20.57 ± 2.90 (range 15-24), and mean grip strength was 60.9% ± 7.4% of the contralateral side. Postoperative wrist mobility assessment revealed the following mean ranges: flexion measured 28.2° ± 7.0°, extension 20.7° ± 6.1°, radial deviation 10.1° ± 3.3°, and ulnar deviation 10.0° ± 4.4°. Forearm rotation was well preserved, with supination reaching 45.4° ± 14.7° and pronation 49.6° ± 10.5° (Table 2). Patients with Grade II tumors had better supination (51.9 ± 10.3 vs. 36.7 ± 16.7, p=0.03) and pronation (53.8 ± 8.9 vs. 43.3 ± 10.3, p=0.04) and higher grip strength (63.8 ± 6.2 vs. 56.8 ± 7.3, p=0.03) than Grade III.

**Table 2:** Postoperative functional outcomes and complications following wide resection. MSTS: Musculoskeletal Tumor Society

S.No.	MSTS Score	Wrist Flexion (degrees)	Wrist Extension (degrees)	Radial deviation (degrees)	Ulnar deviation (degrees)	Supination (degrees)	Pronation (degrees)	Grip strength (%age of uninvolved side)	Complications
1	22	35	25	10	15	0-40	0-45	55	
2	23	35	30	15	10	0-50	0-55	54	
3	17	25	15	10	10	0-35	0-30	48	Fibulo-carpal subluxation Fibulo-ulnar diastasis
4	24	20	15	8	15	0-65	0-60	68	Fibulo-carpal subluxation Fibulo-ulnar diastasis
5	22	30	10	10	15	0-55	0-50	70	Fibulo-carpal subluxation Foot drop
6	24	40	25	12	10	0-60	0-65	74	
7	23	25	15	10	10	0-55	0-55	62	Fibulo-carpal subluxation
8	22	35	25	15	10	0-50	0-60	68	
9	18	20	25	10	5	0-45	0-55	60	Non-union
10	15	20	20	5	0	0-30	0-45	54	Fibulo-carpal subluxation Fibulo-ulnar diastasis
11	19	25	20	5	15	0-60	0-50	58	Fibulo-carpal subluxation Fibulo-ulnar diastasis
12	20	35	30	10	10	0-45	0-55	62	Fibulo-carpal subluxation
13	17	30	15	10	5	30	30	65	
14	22	20	20	7	10	40	35	55	

Complications

**Figure 4:**
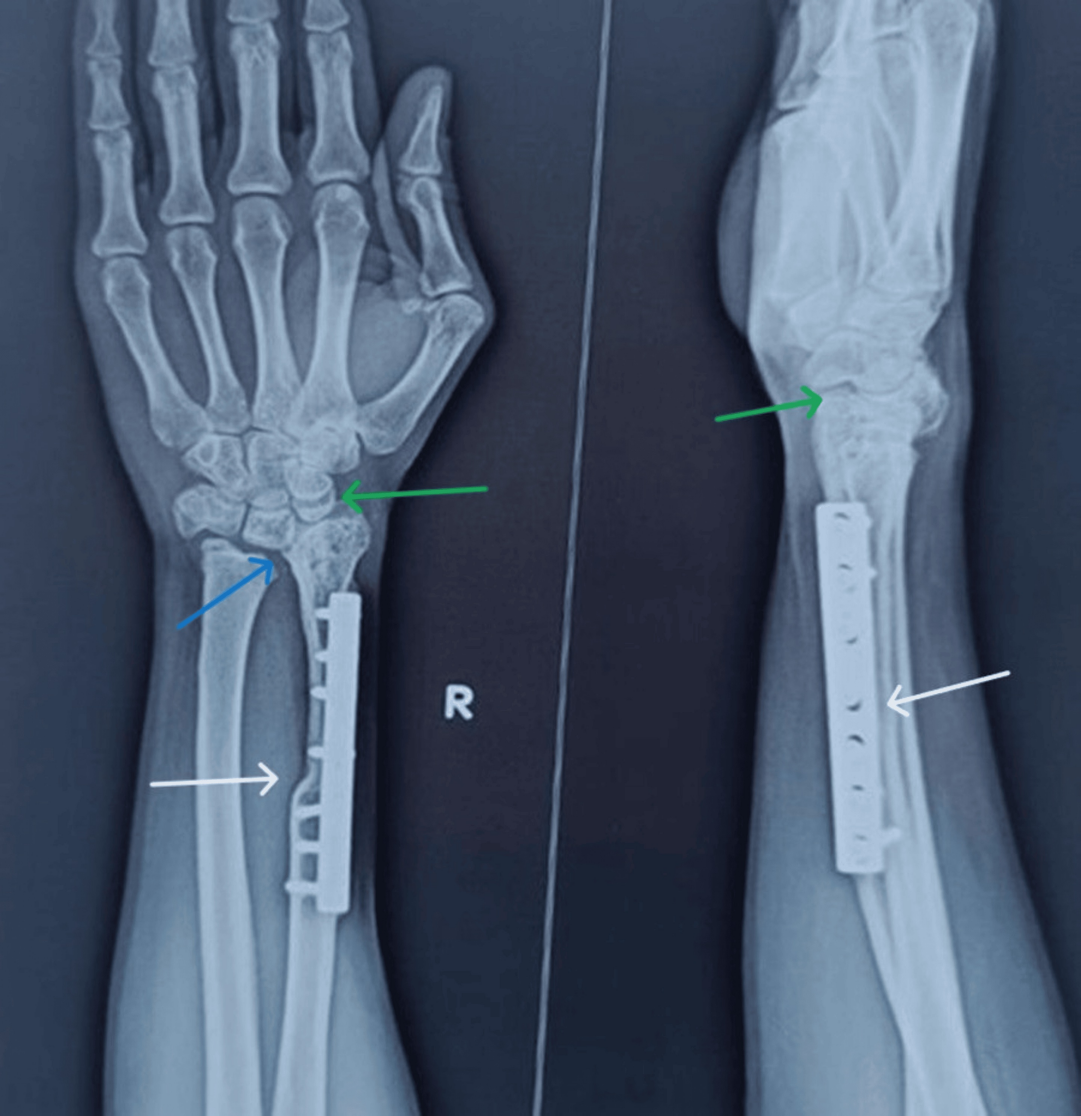
Follow-up imaging Shows solid union of the grafted fibula (white arrows) despite fibulo-carpal subluxation (green arrows) and fibulo-ulnar diastasis (blue arrow).

One patient had a non-union (7.1%) that was successfully treated with iliac crest bone grafting, and there was one instance of transient foot drop (7.1%) that completely resolved with ankle-foot orthosis use. Fibulo-carpal subluxation occurred in seven patients (50%), while fibula-ulnar diastasis was noted in four cases (28.6%) (Figure 4). Significantly worse MSTS scores (19.1 ± 2.9 vs 22.5 ± 1.4, p=0.01) and weaker grip strength (58.1 ± 6.4 vs 64.8 ± 6.5, p=0.03) were noticed in patients who experienced complications (n=eight) compared to those without any complications (n=6), while there was no significant difference in ROM. There was a slight positive correlation between follow-up duration and MSTS score, suggesting that function may improve over time, but this was not statistically significant. No cases of local recurrence or pulmonary metastasis were observed during follow-up.

Return to daily activities

All patients achieved independence in activities of daily living (ADLs), including personal hygiene (bathing, dressing), household tasks (cooking, cleaning), and grooming activities. At final follow-up, 50% of patients (seven) were pain-free, 28.5% (four) had mild and occasional pain, 21.5% (three) had moderate but tolerable pain, and no patient had intolerable pain. Seven patients were manual laborers; four of them returned to their original occupation, while three required job modification due to activity-related pain. Five were homemakers, one was a teacher, and one was a community health worker: all resumed their pre-op activities.

## Discussion

Our findings demonstrate that en-bloc resection with non-vascularized proximal fibular autograft reconstruction is an effective treatment strategy for grade II and III GCT of the distal radius, achieving local disease control in all 14 cases while maintaining clinically meaningful wrist function, as evidenced by the MSTS scores and satisfactory range of motion. The observed complication profile, including graft-related issues and joint instability, reflects known technical challenges of this reconstruction method [9-11], yet did not preclude satisfactory functional recovery in daily activities.

The surgical approach to distal radius giant cell tumors is guided by the Campanacci classification: grade I lesions typically undergo extended curettage with adjuvants, while grade II/III tumors generally require wide resection with reconstruction (arthroplasty or arthrodesis) [13-15]. The non-vascularized ipsilateral fibular autograft offers multiple benefits in the form of faster incorporation due to its autogenous nature, simpler surgical technique compared to vascularized grafts, minimal donor-site morbidity, and partial articular congruity of the grafted fibular head with the carpal bones [16]. Allograft reconstruction presents practical constraints due to limited tissue bank availability and potential immunogenicity. While vascularized fibular grafts may enhance osseous integration, they require microsurgical expertise, extended operative duration, and specialized institutional resources [17, 18].

The range of motion (ROM) required for upper extremity activities of daily living (ADLs), including perineal care, drinking from a cup, reaching the back pocket, and donning/zipping pants, was evaluated by Gates et al. [19] in a motion capture study of 15 participants. They found that these tasks required 38° wrist flexion, 40° extension, 38° ulnar deviation, and 28° radial deviation. In our cohort, ROM following fibular reconstruction for distal radius GCT was lower than this range but comparable to published data, though with some variability. Wrist motion varied across studies, with extension ranging from an average of 31° [4] to 67.3° [20], while it was lower in our study (20.7°), whereas flexion was consistent (31.2°-42° [4, 16, 20, 21]; ours: 28.2°). Radial deviation was similar (10.2° in ours vs 12°-14.1° [16, 20]), but ulnar deviation was lower in our study (10° vs. 19.4°-22° [16, 20]). Supination varied widely (15.3° [20] vs. 52°-57.3° [4, 16, 21]; ours: 45.4°), while pronation ranged from 33.8° [20] to 63.6° [21] (ours: 49.6°). Grip strength was consistently preserved at 60-75% of the contralateral side in most studies [4, 16, 20], with our cohort mirroring the higher end (73%, similar to Lin et al. [20]), while Singh et al. [11] reported lower strength (48.07%).

MSTS functional scores demonstrated remarkable consistency across studies: our mean of 20.57±2.90 (range 15-24) closely matched Barik et al.'s [21] 21.09 (18-24), while Lin et al. [20] and Saini et al. [4] reported better scores of average 25.5 and 91.38% (≈27.4/30), respectively. The majority of our patients (78.5%) were either pain-free or had mild pain, while only three patients (21.5%) had moderate pain, with none having intolerable pain. These findings indicate that non-vascularized fibular reconstruction provides predictable functional results, with variations likely reflecting differences in surgical technique, rehabilitation protocols, or tumor characteristics.

Wrist instability following reconstructive surgery is a well-documented complication, often affecting the fibulo-carpal joint, fibulo-ulnar joint, or both, with reported incidence rates ranging from 10% to 62.5% [22]. Saikia et al. [16] reported fibulo-carpal subluxation in eight cases, with only two requiring treatment due to pain and functional impairment, and Lin et al. [20] reported five cases of wrist dislocation. In the present study, fibulo-carpal subluxation was observed in seven patients, though none experienced complete dislocation, and fibulo-ulnar diastasis was noted in four cases. Importantly, these subluxations were not severe enough to cause significant symptoms or warrant additional intervention, allowing patients to retain a functional range of motion. Although all participants could perform essential activities of daily living-such as cleaning, bathing, cooking, and personal hygiene-without major difficulty. Among the seven patients engaged in heavy manual labor, four successfully returned to their previous jobs, while three had to switch occupations due to pain during prolonged work. The remaining seven patients, whose work was confined to household tasks, adapted well without major limitations.

These findings suggest that while post-surgical wrist instability-particularly subluxation-is relatively common, it often remains clinically asymptomatic. However, patients with physically demanding jobs may experience functional limitations, emphasizing the need for careful postoperative assessment and tailored rehabilitation.

The study further underscores fibulo-radial non-union as a significant complication in wrist reconstruction, with prior studies reporting varying incidence rates: Murray et al. [23] observed five non-unions in 18 cases, Dhammi et al. [24] reported five in 16 cases, Lackman et al. [25] documented two in 12 cases, and Saraf et al. [26] identified five in 15 cases. In contrast, our series demonstrated only one case of non-union, which was successfully treated with iliac crest cancellous bone grafting.

Regarding recurrence, studies [16, 20, 21], Saikia et al. Barik et al. and Lin et al. each reported one case, whereas the present study observed no recurrences over a follow-up period of 13 to 71 months. Additionally, one case of foot drop occurred, which was managed conservatively with orthosis, and the patient had a complete recovery at two months similar complication was noted by Barik et al. as well.

However, the study has several limitations, including its retrospective design, small sample size, and absence of a control group. These factors restrict the generalizability of the findings. To strengthen the evidence, a randomized controlled trial comparing different treatment modalities would be beneficial in determining the most effective surgical and rehabilitation approaches for minimizing complications such as non-union, instability, and nerve injuries. Such a study could provide more definitive guidelines for optimizing patient outcomes.

## Conclusions

En-bloc resection and proximal fibular autograft reconstruction is a viable treatment for distal radius GCT, providing acceptable function despite complications (e.g., non-union, instability). It avoids the use of prostheses or bone banks, making it practical in resource-limited settings. The majority of patients regain the ability to perform daily activities, and many can even return to manual labor, though some may require job modifications. While retrospective data show promise, prospective trials comparing fibular autografts to other treatment methods are needed. Nevertheless, it remains a durable biological solution with satisfactory outcomes.
